# Targeting Malaria Hotspots to Reduce Transmission Incidence in Senegal

**DOI:** 10.3390/ijerph18010076

**Published:** 2020-12-24

**Authors:** Kankoé Sallah, Roch Giorgi, El-Hadj Ba, Martine Piarroux, Renaud Piarroux, Badara Cisse, Jean Gaudart

**Affiliations:** 1 Sciences Economiques et Sociales de la Santé et Traitement de de l’Information Médicale (SESSTIM), Institut de Recherche pour le Développement (IRD), Institut National de la Santé et de la Recherche médicale (INSERM), Aix Marseille Université, 13005 Marseille, France; roch.giorgi@univ-amu.fr (R.G.); jean.gaudart@univ-amu.fr (J.G.); 2 Assistance Publique-Hôpitaux de Paris, Hôpital Bichat Claude Bernard, 75018 Paris, France; 3 Center for Methodology and Modeling, Lomé BP 80956, Togo; 4 Assistance Publique-Hopitaux de Marseille, Hopital La Timone, BioSTIC, Biostatistic and ICT, 13005 Marseille, France; 5 Institut de Recherche pour le Développement (IRD), Université Cheikh Anta Diop, Vecteurs-Infections Tropicales et Méditerranéennes (VITROME), Dakar CP 18524, Senegal; el-hadj.ba@ird.fr (E.-H.B.); badara.cisse@orange.sn (B.C.); 6 Institut Pierre-Louis d’Epidémiologie et de Santé Publique, Institut National de la Santé et de la Recherche médicale (INSERM), AP-HP, Hôpital Pitié-Salpêtrière, Sorbonne Université, 75646 Paris CEDEX 13, France; mpiarroux@yahoo.fr (M.P.); renaud.piarroux@aphp.fr (R.P.)

**Keywords:** malaria elimination, mathematical model, human mobility, intervention chemotherapy

## Abstract

In central Senegal, malaria incidence declined in response to scaling-up of control measures from 2000 to 2010 and has since remained stable, making elimination unlikely in the short term. Additional control measures are needed to reduce transmission. We simulated chemoprophylaxis interventions targeting malaria hotspots using a metapopulation mathematical model, based on a differential-equation framework and incorporating human mobility. The model was fitted to weekly malaria incidence from 45 villages. Three approaches for selecting intervention targets were compared: (a) villages with malaria cases during the low transmission season of the previous year; (b) villages with highest incidence during the high transmission season of the previous year; (c) villages with highest connectivity with adjacent populations. Our results showed that intervention strategies targeting hotspots would be effective in reducing malaria incidence in both targeted and untargeted areas. Regardless of the intervention strategy used, pre-elimination (1–5 cases per 1000 per year) would not be reached without simultaneously increasing vector control by more than 10%. A cornerstone of malaria control and elimination is the effective targeting of strategic locations. Mathematical tools help to identify those locations and estimate the impact in silico.

## 1. Introduction

Malaria remains a major health burden, with a global annual incidence of 228 million new cases and 405,000 deaths in 2018, most of which have occurred in sub-Saharan Africa [1]. In line with the situation in Senegal nationwide, malaria incidence has declined in the Mbour area since the 2000s, due to scaling-up of malaria control. This is primarily due to universal coverage of long-lasting insecticide-treated bednets (LLIN) [2], improved access to diagnosis (Rapid Diagnostic Tests RDT) and prompt treatment of malaria with Artemisinin-based Combination Therapy (ACT) [3,4]. Senegal is still in the control phase of the malaria program, according to the World Health Organization (WHO) classification (more than 5 cases per 1000 inhabitants per year), but the country has been committed to achieving the objectives of pre-elimination by 2020 [5].

Malaria control and elimination projections are challenging due to the complex interactions between humans, vectors, parasite genetic complexity as well as environmental and socioeconomic factors. Spatial heterogeneity characterizes low-transmission settings within non-endemic areas of sub-Saharan Africa and Asia [6,7]. Hotspots are broadly defined as areas where malaria transmission exceeds an average level [8,9]. Targeting interventions to specific hotspots, may be efficient in reducing the burden of malaria in the entire area [8,9,10,11]. Operational definitions of hotspots allow the evaluation of the impact of intervention strategies in dry or rainy seasons. Intervention strategies simulated in this study were:

- Focused Mass Drug Administration (MDA), consisting of systematically treating individuals in a selected geographic area with antimalarial drugs, without screening for infection.
- Focused Mass Screen and Treat (MSAT), consisting of malaria screening, using a rapid diagnostic test and providing treatment to those with a positive test result, in a selected area.
- Seasonal Malaria Chemoprevention (SMC), consisting of intermittently administrating preventive antimalarial treatment to children during the main transmission period.
- Long-Lasting Insecticide-treated Nets (LLIN), intended to avoid mosquito bites, relying on physical and chemical barriers of manufactured nets.

Mathematical modeling consists of describing a phenomenon and using mathematical concepts in order to better understand, control or predict it. Depending on the framework, the models can be predominantly mechanistic or stochastic. Compartmental models are mechanistic models that assign the population to compartments or states, between which individuals may progress. The use of compartmental models in malaria transmission dates back over a century [12] and has made it possible to better understand the malaria transmission and to estimate the effectiveness of control strategies [13,14]. In Senegal, Smith et al. used a stochastic approach to study malaria endemicity in Dielmo and Ndiop [15]. Laneri et al. modeled the impact of climate and immunity on seasonal dynamics [16] and Slater et al. modeled the effect of ivermectin as a new potential vector-control tool to reduce malaria transmission [17].

When several subpopulations are studied simultaneously, one can define a metapopulation model. A metapopulation is made up of a group of spatially separated subpopulations that interact with each other [12]. To date, in Senegal, no study has deployed a metapopulation model in order to obtain global-scale estimates, while simultaneously considering the complex factors that affect the countrywide effectiveness of malaria interventions, including geographical targeting and human mobility. Human mobility may play a critical role in malaria elimination strategies, leading to reintroduction and resurgence of malaria in treated areas, hampering malaria elimination efforts [18].

This study aims to understand the impact of spatially targeted malaria interventions, considering human mobility and using a metapopulation mathematical model based on a susceptible-exposed-infected-recovered (SEIR) framework, with 45 spatially separated villages that interact with each other via moving individuals.

## 2. Materials and Methods

### 2.1. Study Area and Dataset

**Figure 1:**
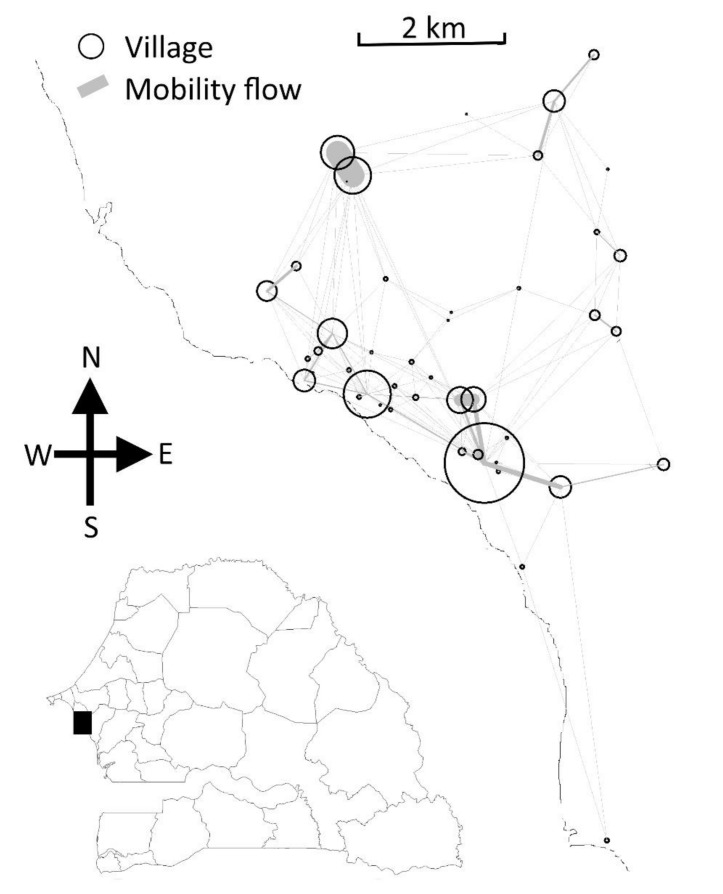
Mbour zone, Senegal, 2008–2012. The geographical coordinates of the 45 villages are represented by black circles, and moving individuals by gray lines. The thickness of the lines reflects the number of trips.

The population data came from 45 villages in the health district of Mbour, Senegal Figure 1 and were collected from 2008 to 2012 through a health and demographic surveillance system established in central Senegal [19]. Malaria cases at health facilities were confirmed by using a rapid diagnostic test and geographical coordinates of village centroids recorded using GPS (Global Positioning System) devices. Estimates of rainfall were extracted from Goddard Earth Sciences Data and Information Services Center. The model was implemented using R 3.1.2 free software. [20], deSolve [21] and FME [22] packages for numerical solution of differential equations describing transmission. The Geosphere R package [23] was used to estimate distances between villages. Graphics were edited with Paint.NET (Rick Brewster, Washington, DC, USA). The dataset analyzed during the current study is available as an additional file.

### 2.2. Model Structure

**Figure 2:**
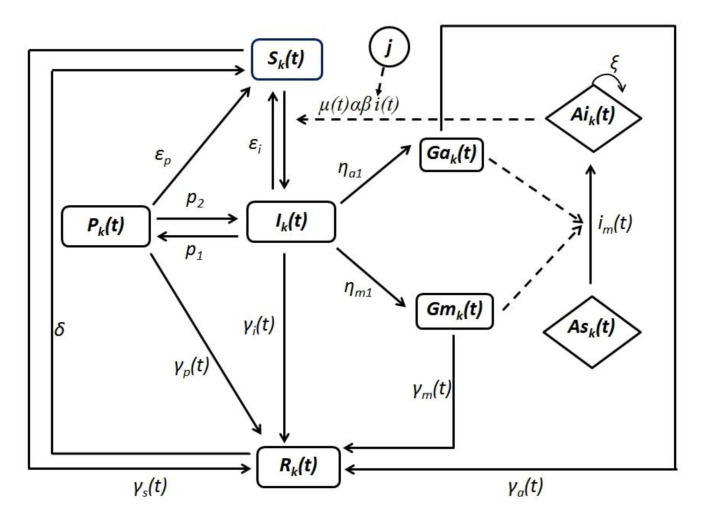
Malaria transmission diagram at a local village k. Letter *j* stands for remote villages. Human compartments are $S_{k}$ (susceptible), $P_{k}$ (premunition), $I_{k}$ (blood-stage infection), $Ga_{k}$ (asymptomatic carriage of gametocytes), $Gm_{k}$ (symptomatic carriage of gametocytes) and $R_{k}$ (resistance due to treatment). Mosquito compartments are $Ai_{k}$ (infected mosquitoes) and $As_{k}$ (susceptible mosquitoes). The arrows represent the transition rates between compartments.

Malaria transmission in each village was represented by a deterministic compartmental SEIR transmission model, based on the “Bancoumana” model described by Gaudart et al. [13], as seen in Figure 2. At the time *t*, individuals from the susceptible compartment $S_{k}\left(t\right)$ may get infected. The proportion of human infection in village k, denoted $I_{k}\left(t\right)$, was proportional to anopheles density υ(t), to frequency of mosquito bites α, to human susceptibility to infection β and to the effective proportion of infected mosquitoes i(t). The latter represent a weighted sum of the local proportion of infected mosquitoes $Ai_{k}\left(t\right)$ and the remote proportion of infected mosquitoes $Ai_{j}\left(t\right)$ Equation (1). The weights depended on the proportion (m) of people that are away at a given time, and also on relative probabilities $Q_{kj}$ of travel from remote locations *j* to local village *k*.

(1) $$i\left(t\right)=\left(1-m\right)Ai_{k}+m\underset{j\neq k}{\sum }Q_{kj}Ai_{j}$$

Probabilities *Q_kj_* were estimated via the radiation model of human mobility [24] and is represented here as Equation (2):

(2) $$Q_{kj}=\frac{P_{k}P_{j}}{\left(P_{k}+s_{kj}\right)\left(P_{k}+P_{j}+s_{kj}\right)}$$

In Equation (2), $P_{k}$ and $P_{j}$ are the population sizes in locations *k* and *j*, respectively and $s_{kj}$ is the total population inside the circle centered at *k*, whose circumference touches *j*, excluding the source and destination populations ($P_{k}$ and $P_{j}$). Travel was modeled as round trips of approximately a one-week duration. The population sizes of the villages were updated annually over the study period. Each inhabitant of a village *k* could infect or be infected at other villages *j*. In this approach, moving individuals remain residents of their home village, but spend some time in neighboring villages. Long-term mobility is not incorporated.

The model assumes that newly infected individuals at village *k*, $I_{k}\left(t\right)$, initially carry only blood-stage infection. Gametocytes are sexual precursor cells of the malaria parasite that mediate the transmission of the parasite from the host to the Anopheles mosquito. Gametocytes subsequently appear and the individual may have malaria symptoms or stay asymptomatic, leading to $Gm_{k}$ (symptomatic, infectious) and $Ga_{k}$ (asymptomatic, infectious) compartments, respectively. All gametocyte carriers were assumed to contribute to transmission. Infection of mosquitoes depends on the effective proportion of human infection $i_{m}\left(t\right)$, represented as a weighted sum of human infection at local and neighboring villages. Gametocytes were transmitted to anopheles from gametocyte carriers that reside in the local village *k* and by gametocyte carriers that travel from remote villages *j* to the local village *k*. It was assumed that mosquitoes are infected by feeding on humans carrying gametocytes and that blood-fed anopheles do not move from one village to another [25].

The model assumes that adults gradually acquire partial immunity (premunition) at a rate $p_{1}$ [26], after several malaria attacks. Premunition is assumed to be lost at rate $p_{2}$ [26].

Targeted interventions were modeled as a transition of individuals to the resistant compartment. The transition rates are defined as rectangular pulse functions reflecting interventions, over a limited period of time. The protection resulting from drug administration is assumed to be lost at a constant rate depending on the antimalarial half-life.

**Figure A1:**
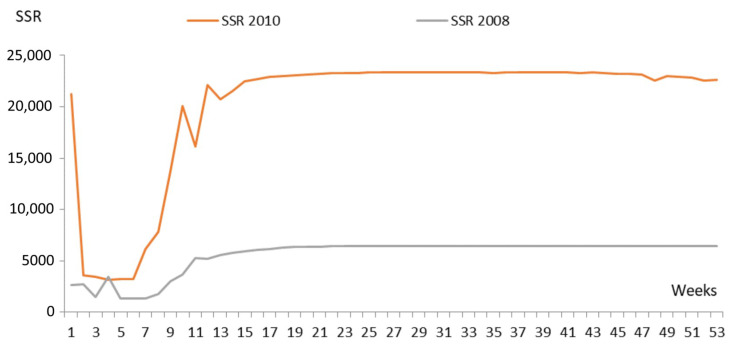
Goodness of fit of malaria metapopulation model according to the assumed lag between rainfall and mosquito bites. Calibrations were undertaken on 2008 and 2010 transmission seasons. Optimization function computing the sum of squared residuals (SSR) were minimized for 6 weeks lag.

The seasonal variations of anopheles density, υ(t), were modeled assuming that the anopheles density is proportional to the cumulative rainfall over the previous six weeks and oscillated seasonally between the minimum and maximum values reported in previous entomological studies for this area (0 to 12 anopheles/individual/day) [27]. The correlation lag between the density of anopheles and the malaria incidence was estimated by sensitivity analysis, and can be seen in Figure A1. The estimated value was about 6 weeks, 95% CI (3–8 weeks). This value was consistent with previous studies [28,29].

The equations of the model are set out in Appendix A, and a description of the parameters is given in Table A1.

### 2.3. Model Calibration

The metapopulation model was fitted to weekly malaria incidence data from 1 January 2008, to 31 December 2008, using an optimization approach based on the Markov Chain Monte Carlo (MCMC) method [30].

Initial values of the compartments were defined at the beginning of each rainy season. Several parameter values relied on values from the literature [31] Table A1 and Table A2. The sensitivity of the parameters was assessed by varying them around the estimated value.

### 2.4. Hotspots Definitions and Interventions

Three pragmatic definitions of hotspot were investigated:

1. Low transmission period hotspots (LT hotspots) were defined as villages reporting at least one malaria case in the previous low transmission period (December to May).
2. High transmission period hotspots (HT hotspots) were villages with the highest malaria incidences during the previous transmission season (June to November).
3. High connectivity hotspots (HC hotspots) were villages highly connected to neighboring villages based on human mobility potential.

Connectivity was approximated by the degree centrality score Equation (3). Degree centrality of village *k* ($d_{k}$) was defined as the number of travel connections from outside villages to village *k* and which volumes were above the first decile of total volume of travels towards *k* [32]. Degree centrality captures infection routes from outside villages to *k* and higher values indicate an increased vulnerability to malaria spread.

(3) $$d_{k}=card(w_{jk}|w_{jk}\geq 0.1w_{k})$$

In Equation (3), $d_{k}$ represents the degree centrality score of village *k*, *card* (cardinality) represents the number of connections to village *k* above the threshold of 10%, $w_{jk}$ represents the number of trips from villages *j* to village *k* and $w_{k}$ represents the total volume of travel to village *k.*

These definitions were kept deliberately simple to be applicable in practice and require neither prior serological surveys nor special clustering analysis [8,10,33].

In silico interventions were simulated from 2010. MSAT and MDA drug interventions assumed the use of dihydroartemisinin-primaquine. Benefits of Artemisinin compounds include rapid parasite clearance, but, when used alone, recrudescence rates are high. Primaquine has a potent gametocidal effect, meaning it can help block transmission. The coverage rate was set to 70% for each round of MDA/MSAT, meaning that 70% of the population in targeted hotspots effectively received the intervention (treatment in the case of MDA and pre-treatment screening in the case of MSAT). Two rounds of intervention, separated by a one-month interval, were assumed for both MDA and MSAT, with drugs provided during the first week of September and again during the first week of October (high transmission period simulations) or in February and March (low transmission period simulations).

The simulated SMC strategy assumed the use of sulphadoxine-pyrimethamine-amodiaquine, and targeted only children under 10 years old, representing 30% of the population [34]. Delivery occurred on the first 4 days of each month from September to December, in the entire study area. According to WHO recommendations, SMC should not be implemented as a geographically limited targeted strategy. The impact of long-lasting insecticidal nets was implemented as a direct decrease in the rate of mosquito bites (α) over the intervention period.

The intervention efficacy, $\Delta_{I}$ was defined as the relative variation in malaria annual incidence from no intervention assumption to intervention assumption:

(4) $$\Delta_{I}=1-\frac{I_{1}}{I_{0}}$$

In Equation (4), *I*_0_ and *I_I_* were the cumulative incidences of malaria, respectively before and after intervention. 

## 3. Results

### 3.1. Parameters Estimates and Sensitivity Analysis

The estimated weekly mobility rate was m = 0.09 (95% CI: 0.0015–0.2) corresponding to 2–200 individuals moving between villages per 1000 inhabitants per week. The entomological inoculation rate (EIR), calculated from the model, varied seasonally between 0 and 2.16 infected bites per person per night. This was consistent with literature data [4].

**Figure 3:**
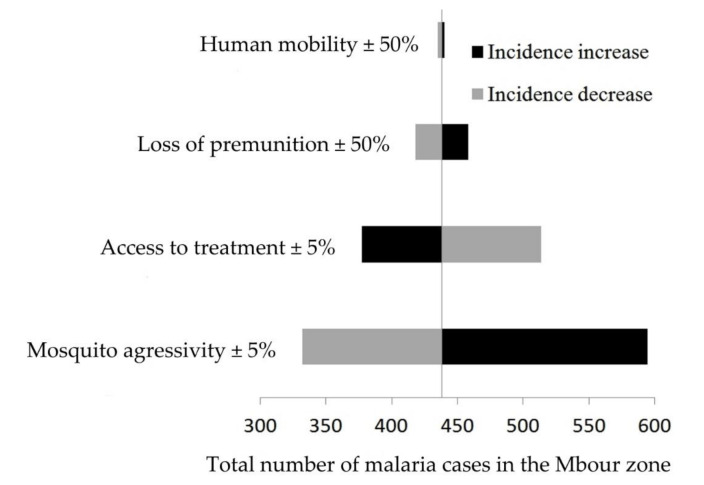
Sensitivity of model parameters in the malaria metapopulation model, Mbour, Senegal, 2008–2012. Right and left correspond to a parameter increase and decrease, respectively. Black and gray bars respectively represent a increase and decrease in total malaria cases, subsequent to parameter variations.

Key parameters were varied to assess their sensitivity on malaria incidences Figure 3. Model predictions were sensitive to the following parameters: density of anopheles (a 33% increase in malaria incidence while increasing the parameter by about 5%), access to treatment (a 16% increase in malaria incidence while decreasing the parameter by about 5%), loss of premunition (a 4.5% increase in malaria incidence for a 5% parameter increase) and human mobility (a 1% increase in malaria incidence for a 100% parameter increase).

### 3.2. Sensitivity of Hotspot Definitions

LT hotspots showed temporal instability. Their locations changed from one year to another (Cohen’s Kappa coefficient 0.21, 95% CI: 0.16–0.33 versus 0.6, 95% CI: 0.36–0.85 for HT hotspots). HC hotspots were almost static in time, because this definition relied on mobility estimates, based on population densities, while relative variations remained comparable between sites.

HT hotspots were less populated than LT hotspots (average population per HT hotspot, with 510 inhabitants in HT hotspots versus 1703 inhabitants in LT hotspots, Wilcoxon test *p* = 0.13), suggesting that small population groups had higher incidence rates during the transmission season.

HC hotspots were slightly more populated than LT hotspots (average population per HT hotspot, with 1876 inhabitants in HT hotspots versus 1703 inhabitants in LT hotspots, Wilcoxon test *p* = 0.6) and demonstrated lower malaria incidences than LT hotspots (Wilcoxon test *p* = 0.03).

### 3.3. Intervention Simulations

**Figure 4:**
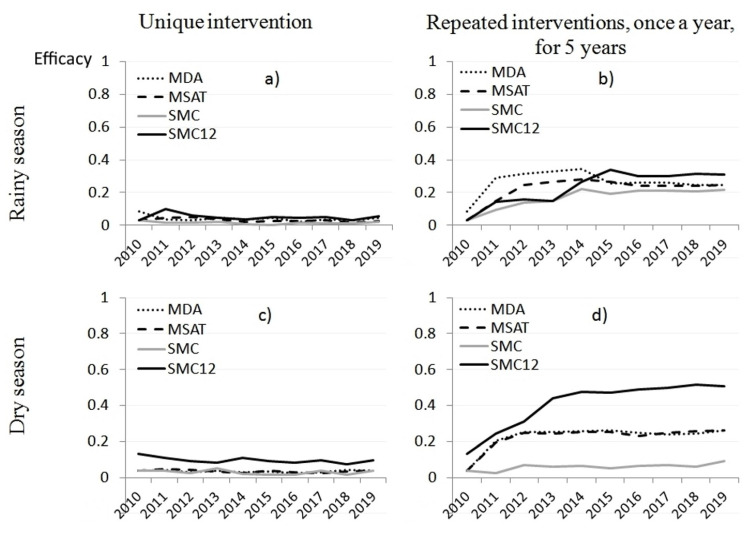
Decrease in malaria incidence while targeting low transmission (LT) hotspots in Mbour, Senegal, 2008–2012. The y-axis represents the relative decrease in malaria incidence for the overall area (45 villages). (**a**) unique one-year intervention in the rainy season, (**b**) repeated interventions over five consecutive rainy seasons, once per year, (**c**) unique one-year intervention in the dry season, (**d**) repeated interventions over five consecutive dry seasons, once per year. SMC12 corresponds to a theoretical schedule of uninterrupted monthly administration of SMC over 12 months.

Variations in annual incidences after a unique intervention and after yearly repeated interventions on LT hotspots are shown on Figure 4, for the overall study area.

Percentage of villages defined as LT hotspots in 2011 and 2012 were 35% and 31%, respectively. As LT hotspots were not predictable beyond data limits, we assumed that their proportion would remain at 31%, in order to allow forecasting. Repeating MDA and MSAT interventions in LT hotspots, once per year, during the rainy seasons, after five consecutive years, yielded a decrease in malaria incidence of 34% and 28%, respectively. As interventions stopped, the efficacy reverted and stabilized at 25%. After delivering SMC sequentially in dry seasons, efficacy reached only 10% after 5 years. Effects were higher after SMC was sequentially delivered in rainy seasons (20% after 5 years). Monthly uninterrupted SMC would reach a 50% incidence decrease after 5 consecutive years.

When targeting the equivalent proportion of HT hotspots, repeated interventions stabilized at 56% efficacy when delivered during the dry season. When delivered during the rainy seasons, they respectively yielded 67% and 56% long-term efficacy (Figure 2).

Targeting equivalent proportion of villages according to HC hotspot definitions, five years of repeated interventions during the rainy seasons yielded 74% and 64% efficacy, respectively, for MDA and MSAT, which decreased and stabilized at 57% cessation of interventions.

### 3.4. Pre-Elimination/Elimination Stage

**Figure 5:**
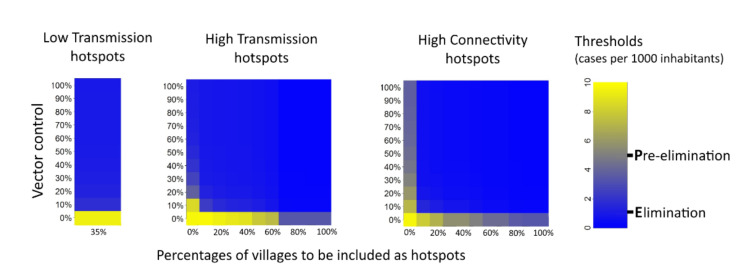
Malaria incidence in the year following mass drug administration associated with vector control. Various definitions of hotspots were tested. The x-axis represents the percentage of villages included as hotspots. The y-axis represents the decrease in mosquito bites from baseline.

MDA simulated over one single year, targeting LT hotspots, led to the pre-elimination stage (1–5 cases per 1000 per year), provided that mosquito bites were simultaneously reduced by 10% or more, as can be seen in Figure 5. The elimination stage (less than 1 case per 1000 per year) would be theoretically achievable by combining a 70% vector decrease and MDA in LT hotspots. When targeting HT or HC hotspots, more than a 10% simultaneous decrease in mosquito bites would be needed to reach pre-elimination, regardless of the MDA coverage.

### 3.5. Rebound Effects Due to Human Mobility

An incidence rebound (incidence increase after interventions ended) was noticed at the cessation of repeated MDA/MSAT interventions. Rebounds occurred only if mobility was considered to not be null. While targeting one third of HC hotspots for five consecutive rainy seasons, rebound was about 17% for the overall area and 43% in targeted villages. This may be a worst-case scenario, as we assumed an average proportion of 20% travelers would be moving between villages.

## 4. Discussion

This study investigated the use of a spatially explicit malaria metapopulation model, fitted to weekly malaria incidence in rural villages in central Senegal.

A final decrease in the incidence of malaria, of more than 25% was reached in the overall area, with both MDA and MSAT simulated interventions repeated for five years on LT hotspots and 57% on HT or HC hotspots. Monthly uninterrupted SMC simulated on the 45 villages over five years showed similar results (a 50% decrease in the incidence). Reaching the pre-elimination stage (1–5 cases per 1000 per year) was possible only when simultaneously decreasing mosquito bites by more than 10%. We highlighted the foreseeable interest of spatially targeted interventions.

Obviously, the reservoir of parasites is not limited to hotspots. The asymptomatic reservoir in untargeted areas may trigger transmission, especially when mosquito bites increased at the beginning of a new rainy season. This would explain why targeting LT hotspots (31–35% of villages, supposed to be the bottleneck in the dry season) was not enough to reach the elimination stage, despite the important impact of this strategy. Targeting LT hotspots in the dry season was intended to quickly clear the parasite reservoir when its level was low. But if a widespread asymptomatic parasite carriage is assumed, high coverage and repeated interventions would be needed to reach elimination. Asymptomatic and sub-microscopic parasite carriage should be investigated to display geographical patterns of the reservoir [35,36]. Further research is needed on the relationship between sub-microscopic parasitemia and clinical malaria hotspot definition [37]. It has been argued that clinical malaria incidences should not be used in hotspot definitions without considering asymptomatic malaria patterns [8,38] and clustering of asexual parasite carriage, using serological tools to detect malaria-specific immune responses [8].

Human mobility has usually been identified as a threat to malaria-free areas [14,39]. In our study, malaria incidence decreased in untargeted areas due to the decrease in malaria importation. Some studies assumed that this could be related to fewer infected mosquitoes moving from targeted areas [40], but mosquito mobility modeling was not relevant in our model. More than 80% of the villages were more than 3 km far from the nearest village. Real human mobility data may be more accurate than estimations from the radiation model. However, concerns about the geographical scale prevented us from using proxies, such as anonymized call details records [41], to estimate mobility. Systematic studies are needed to inform mobility patterns in rural and semi-rural malaria areas in Senegal.

In the past, MDA interventions have contributed to eliminating malaria from islands and remote areas, where population movements were closely controlled and gametocytocidal drugs have been used [42,43].

No resistance to dihydroartemisinin-primaquine was previously reported in our study area by 2017, and therefore this was not modeled.

In practice, coverage and efficacy of drug interventions would also depend on the cooperation, involvement and education of local communities, alongside good communication and support from local authorities [44].

## 5. Conclusions

Our metapopulation model specifically and explicitly considered human mobility at the village scale, analyzing malaria transmission and interventions efficacy in Senegal. Regardless of the type of intervention, the pre-elimination stage (1–5 cases per 1000 per year) could not be reached without simultaneously increasing vector control by more than 10%. Compartmental modeling remains an interesting tool to specifically guide malaria strategies and policies. Nevertheless, this deterministic approach needs to be cautiously interpreted. Unexpected changes in climatic, biological and socio-environmental factors could generate high inaccuracies in predictions.

## Figures and Tables

**Figure A2:**
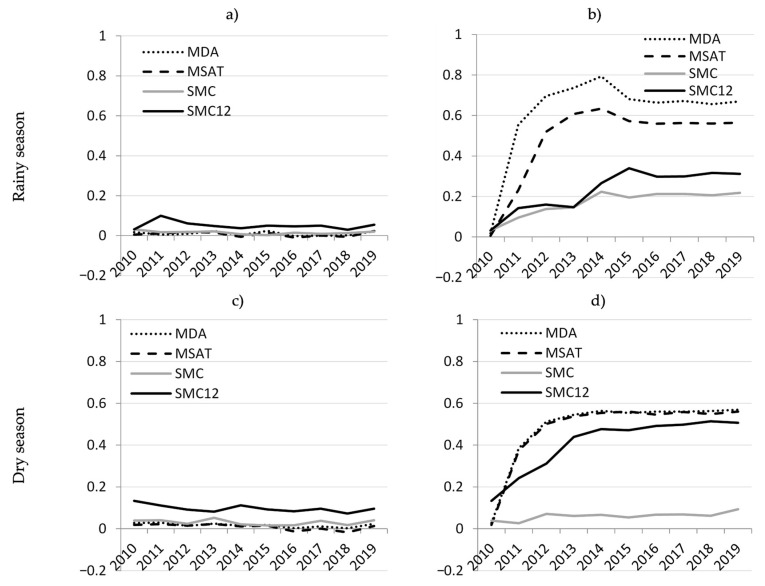
Decrease in malaria incidence while targeting one third of HT hotspots in Mbour, Senegal 2008–2012. The y-axis represents intervention efficacy (relative decrease in overall malaria incidence). (**a**) Unique one-year intervention during rainy season, (**b**) Repeated interventions on five consecutive rainy seasons, once per year, (**c**) Unique one-year intervention during the dry season, (**d**) Repeated interventions on five consecutive dry seasons, once per year.

**Figure A3:**
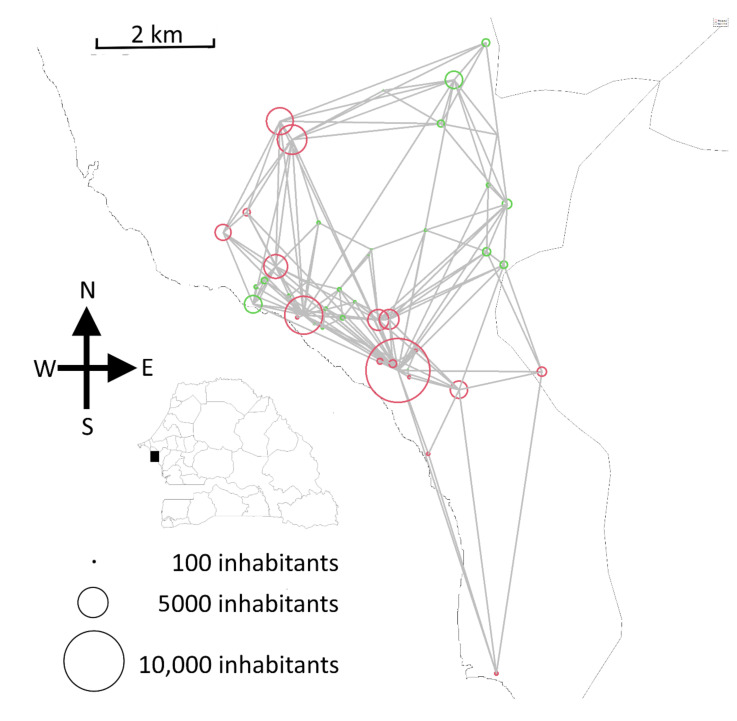
Mbour zone, Senegal, 2008–2012. Geographical coordinates of the 45 villages circles. Red circles represent connectivity hotspots and gray lines represent main connections between villages.

## List of Abbreviations

LLIN	long-lasting insecticide-treated bed nets
RDT	rapid diagnostic tests
ACT	artemisinin-based combination therapy
WHO	World Health Organization
MDA	mass drug administration
MSAT	mass screen and treat
SMC	seasonal malaria chemoprevention
SEIR	susceptible-exposed-infected-recovered
GPS	global positioning system
MCMC	Markov Chain Monte Carlo
LT hotspots	low transmission period hotspots
HT hotspots	high transmission period hotspots
HC hotspots	high connectivity hotspots
HTP	high transmission period
LTP	low transmission period
EIR	entomological inoculation rate
IRS	indoor residual spraying

## Appendix A

### Appendix A.1. Model Description

In each village *k*, malaria transmission was described by a set of equations describing variations in human and mosquito compartments. The terms of the equations are defined below:

- $S_{k}\left(t\right)$: proportion of humans susceptible to malaria infection.
- $I_{k}\left(t\right)$: proportion of blood-stage infected humans, with few gametocytes, not immune and positive to rapid diagnostic test (RDT).
- P(t): proportion of humans with partial immunity (premunition). Individuals could remain in this state for many years, but could lose their immunity if pregnant or on cessation of exposure. They were assumed to be RDT positive and with few gametocytes.
- $Ga_{k}\left(t\right)$: proportion of infected humans, gametocyte-positive, asymptomatic.
- $Gm_{k}\left(t\right)$: proportion of infected humans, gametocyte-positive, symptomatic.
- $R_{k}\left(t\right)$: proportion of humans who were temporarily not susceptible to new infection, as a result of the prophylactic effect of treatment.
- $Ai_{k}\left(t\right)$: proportion of female mosquitoes that carry sporozoites in their salivary glands.
- $As_{k}\left(t\right)$: proportion of female mosquitoes that have survived the cycle and were free from malaria sporozoites.
- m: proportion of people in the overall population, who are away at a given time (visiting other villages than their own village).
- υ(t): anopheles’ density (ratio of the number of female anopheles to the number of humans, at time *t*).
- α: number of bites per female anopheles per night.
- β: probability that a person bitten by an infectious mosquito becomes infected.
- $\gamma_{s}\left(t\right)$, $\gamma_{i}\left(t\right)$, $\gamma_{p}\left(t\right)$ and $\gamma_{a}\left(t\right)$ are rectangular pulse functions. γ(t)=K=−log(1−c)/Δ where c represents coverage and Δ the duration of intervention in weeks.
- $\gamma_{s}\left(t\right)$: rate at which susceptible individuals are treated.
- $\gamma_{i}\left(t\right)$: rate at which blood-stage infected individuals are treated.
- $\gamma_{p}\left(t\right)$: rate at which naturally immune individuals are treated. Naturally immune individuals are assumed RDT positive. Susceptible individuals are RDT negative.
- $\gamma_{a}\left(t\right)$: rate at which asymptomatic gametocyte carriers are treated. Asymptomatic gametocyte carriers are assumed RDT positive.
- $\gamma_{m}\left(t\right)$: rate at which symptomatic gametocyte carriers are treated. This corresponds to access to care, in periods of no intervention.
- $\eta_{a1}$: transition rate from blood-stage infection, to asymptomatic gametocyte carriage.
- $\eta_{m1}$: transition rate from blood-stage infection, to symptomatic gametocyte carriage.
- $p_{1}$: transition rate from blood-stage infection to premunition.
- $p_{2}$: transition rate from premunition to blood-stage infection (loss of premunition).
- *δ*: transition rate from resistant to susceptible (loss of the protection due to treatment).
- $\zeta_{m}$: probability that a mosquito, biting a symptomatic gametocyte carrier, got infected.
- $\zeta_{a}$: probability that a mosquito, biting an asymptomatic gametocyte carrier, got infected.
- ξ: mortality rate of mosquitoes.
- $Q_{kj}$: relative probabilities of travel from remote locations *j*, to local village *k*.
- r(t): rainfall at week *t*.

Differential Equations describing malaria transmission inside a village *k* are listed below. *j* stands for remote villages.

(A1) $$\frac{dS}{dt}=-\upsilon \times \alpha \beta S\times i+\delta R-\gamma_{s}S$$

(A2) $$\frac{dI}{dt}=v\times \alpha \beta S\times i-(\eta_{a1}+\eta_{m1}+\gamma_{i}+p_{1})I+p_{2}P$$

(A3) $$\frac{dP}{dt}=p_{1}I-\;\left(p_{2}+\gamma_{p}\right)P$$

(A4) $$\frac{dGa}{dt}=\eta_{a1}I-\gamma_{a}Ga$$

(A5) $$\frac{dGm}{dt}=\eta_{m1}I-\gamma_{m}Gm$$

(A6) $$\frac{dR}{dt}=\gamma_{m}Gm+\gamma_{a}Ga+\gamma_{i}I+\gamma_{p}P+\gamma_{s}S\;-\delta R$$

(A7) $$\frac{dAi}{dt}=\alpha \left(1-Ai\right)i_{m}\;-\xi Ai$$

(A8) $$i=\left(1-m\right)Ai+m\underset{j\neq k}{\sum }Q_{kj}Ai_{j}$$

(A9) $$i_{m}=\left(1-m\right)\left(\zeta_{a}Ga+\zeta_{m}Gm\right)+m\underset{j\neq k}{\sum }Q_{jk}\left(\zeta_{a}Ga_{j}+\zeta_{m}Gm_{j}\right)$$

(A10) $$\upsilon \propto \mu \overset{t}{\underset{t-lag}{\sum }}r$$

**Equation (A1) describes the variations in the proportion of susceptible individuals.** In each village *k*, individuals leave the susceptible compartment by getting infected. The human infection rate consists of the product of the anopheles density υ, the frequency of mosquito bites α, the human susceptibility to infection β and the effective proportion of infected mosquitoes affecting village *k*, represented by i.

The proportion of susceptible individuals in village *k*, was increased by individuals losing their protection from the resistant compartment (*R*), who were no longer under treatment effect. These individuals entered the susceptible compartment at rate δ. In the case of mass intervention, the proportion of susceptible individuals in village *k* was decreased by the fraction of treated individuals ($-\gamma_{s}\times S$).

Population sizes remained stable, since travel was assumed to be round trips. We updated the population size of each village by year. For the sake of simplification, each year, at the week level, birth and death rates were balanced. Real population growth rate was estimated 0.023% per week [34].

**Equation (A2) describes the variations in the compartment (*I*) of blood-stage infection.** New infections increased the compartment *I* by υ×αβS×i . Compartment *I* was decreased by gametocyte production ($-\left(\eta_{a1}+\eta_{m1}\right)I$) and acquisition of premunition ($-p_{1}I$).

**Equation (A3) describes the variations in the compartment (*P*) of pre-immune.** The compartment increased by receiving individuals acquiring premunition after several blood-stage infections ($+p_{1}I$). The compartment decreased when infection was reactivated by loss of immunity ($-p_{2}P$) or by interventional treatment ($-\gamma_{p}P$).

**Equations (A4) and (A5) describe the variations in the compartments of gametocyte carriers.** Compartments of gametocyte carriers increased by receiving individuals from blood-stage infection ($+\eta_{a1}I$ or $+\eta_{m1}I$). These compartments could decrease by transition to resistant compartment because of treatment ($-\gamma_{a}\times Ga$ ,$\;-\gamma_{m}\times Gm$).

**Equation (A6) describes the variations in the resistant compartment.** This compartment was increased by individuals under treatment effects (usual malaria therapies or interventional drugs), coming from all compartments where an effective malaria treatment had been delivered. This compartment was depleted by the loss of protection (−δR), beyond drug half-life. Long-acting drugs dihydroartemisinin-primaquine and sulphadoxine-pyrimethamine-amodiaquine were used for MDA/MSAT and SMC, respectively, all yielding protection for about 4 weeks’ duration.

**Equation (A7) describes the variations in the proportion of infective mosquitoes.** The proportion of infective mosquitoes was the product of the frequency of mosquito bites (α) and the effective proportion of infected humans ($i_{m}$). The proportion of infective mosquitoes was decreased by deaths in mosquito population ($-\xi Ai_{k}$).

**Equation (A8) details the effective proportion of infective mosquitoes in a location *k* taking account of human mobility.** This proportion is represented by $\;i=\left(1-m\right)Ai_{k}+m\overset{}{\underset{jk}{\sum }}Q_{kj}Ai_{j}$. The effective proportion of infected mosquitoes affecting village *k* is a weighted average of local proportions of infected mosquitoes $Ai_{k}$ and remote proportions of infected mosquitoes ($Ai_{j}$). The weights depended on the proportion of human mobility m and also on the relative probabilities of travel from remote locations *j* to local village *k* ($Q_{kj}$).

**Equation (A9) details the effective proportion of infected humans in a location *k* taking account of human mobility.** This proportion is represented by $i_{m}=\left(1-m\right)\left(\zeta_{a}Ga_{k}+\zeta_{m}Gm_{k}\right)+m\overset{}{\underset{j\neq k}{\sum }}Q_{kj}\left(\zeta_{a}Ga_{j}+\zeta_{m}Gm_{j}\right)$ as a weighted average of the local proportion of infected humans $\left(\zeta_{a}Ga_{k}+\zeta_{m}Gm_{k}\right)$ and remote proportions of infected humans $\underset{j\neq k}{\sum }Q_{kj}\left(\zeta_{a}Ga_{j}+\zeta_{m}Gm_{j}\right)$, weekly. The weights depended on the proportion of human mobility (m) and on the relative probabilities of travel from remote locations *j* to local village *k* ($Q_{kj}$). The susceptibility of mosquitoes to infection from humans ($\zeta_{m}$) was assumed ten times higher from symptomatic than from asymptomatic ($\zeta_{a}$) (expert opinion).

**Equation (A10) represents variations in anopheles’ density with respect to rainfall.** Anopheles density depends on deterministic environmental factors. Among these factors, accumulated rainfall in previous weeks was the most important. *lag* was the duration (in weeks) between rainfall and mosquito bites. Optimal lag was estimated using sensitivity analysis as the one minimizing the gap between model estimations and observations Figure A1.

### Appendix A.2. Model Parameters

In each village *k*, malaria transmission was described by a set of equations describing variations in human and mosquito compartments. The terms of the equations are defined below:

**Table A1:** Model Parameters.

Parameter Symbol	Parameter Description	References	Parameter Values	95% C.I.
υ	Anopheles density in relation to hosts	[27,31]	0–12 (min–max)	
α	Mosquito biting rate	[45]	0.46 bite/anopheles/night	
β	Human susceptibility to infection	[45]	0.3	
EIR	Entomological Inoculation Rate	[46]	0 to 2.16/per person/year	
$\zeta_{m}$	Mosquito susceptibility to infection from symptomatic humans	[45]	0.80	
$\zeta_{a}$	Mosquito susceptibility to infection from asymptomatic humans	Expert opinion	0.08 $(\zeta_{a}=0.1\zeta_{m}$)	
$\eta_{m1}$	Transition rate from blood-stage parasitemia to symptomatic infection with gametocytemia	[47]	0.1 days^−1^	
$\eta_{a1}$	Transition rate from blood-stage parasitemia to asymptomatic infection with gametocytemia	[47]	0.1 days^−1^	
δ	Transition rate from post-treatment protection, to susceptible	[48,49]	0.032 days^−1^	
ξ	Daily mosquito mortality rate	[45]	0.18	
$p_{1}$	Rate of the acquisition of premunition	fitted	0.0002 days^−1^	0.0001–0.00035 days^−1^
$p_{2}$	Rate of the loss of premunition	fitted	0.0002 days^−1^	0.0001–0.00035 days^−1^
$\gamma_{m}$	Usual recovery rate by access to care, not related to specific interventions	fitted	0.37 days^−1^	0.20–0.51 days^−1^
m	Proportion of people who are away from their home village at a given time	fitted	0.01	0.09–0.2

**Table A2:** Initial conditions.

Compartment	Assigned Value	Reference
$I_{0}$	$I_{0}\approx 0$. Proportion of plasmodium falciparum infection in humans, in dry season	[27,45]
$P_{0}$	$P_{0}$ = 0.2, Proportion of pre-immune individuals	0.16 [46] B0.27 [47] 0.23-0.32 [31]
$Gm_{0}$	Proportion of symptomatic malaria in dry season. Average dry season incidence estimated from all the dataset (5years data)	[48]
$Ga_{0}$	Proportion of asymptomatic gametocyte carriers were assumed 10 times lower than symptomatic (expert advice)	$\mathrm{Deduced}\;\mathrm{from}\;Gm_{0}$
$R_{0}$	$R_{0}\approx Gm_{0}$. Continuous access to treatment for symptomatic malaria.	$\mathrm{Deduced}\;\mathrm{from}\;Gm_{0}$
$Ai_{0}$	$Ai_{0}\approx 0.$ Proportion of female anopheles that carry sporozoites, in dry season	[27,45]
$S_{0}$	$S_{0}=1-Gm_{0}-Ga_{0}-R_{0}-P_{0}$	Calculated

## Appendix B

### Appendix B.2. Simulation of Interventions Targeting HT Hotspots

High-connectivity hotspots are defined according to the radiation model Figure A2 directy depend on population sizes and remain almost stable. For other definitions, hotspots change from one year to another (spatio-temporal variations).
